# Reproduction Indicators Related to Litter Size and Reproduction Cycle Length Among Sows of Breeds Considered Maternal and Paternal Components Kept on Medium-Size Farms

**DOI:** 10.3390/ani10071164

**Published:** 2020-07-09

**Authors:** Błażej Nowak, Anna Mucha, Magdalena Moska, Wojciech Kruszyński

**Affiliations:** Department of Genetics, Wrocław University of Environmental and Life Sciences, 51-631 Wrocław, Poland; anna.mucha@upwr.edu.pl (A.M.); magdalena.moska@upwr.edu.pl (M.M.); wojciech.kruszynski@upwr.edu.pl (W.K.)

**Keywords:** sows, reproduction, breed, litter size, farrowing interval, principal component analysis

## Abstract

**Simple Summary:**

Pig breeds considered maternal components are bred mainly to improve reproductive traits, while those considered paternal components are bred to improve production traits. These two groups of traits are either negatively or weakly positively correlated. For any breed, however, the key factors affecting the profitability of pig production are a properly conducted reproduction process and proper herd management. This research compared reproduction indicators related to litter size (litter size along with the numbers and percentages of piglets born alive, stillborn, and weaned) and reproduction cycle length (the lengths of gestation, lactation, the weaning-to-conception interval, and the farrowing-to-conception interval) among sows of maternal (Polish Large White, Polish Landrace, and Yorkshire) and paternal (Duroc, Hampshire, and Berkshire) breeds. The pigs were raised on three medium-sized farms, of which two were located in Poland and one in the US. The results suggest that Polish Large White and Polish Landrace sows, both considered maternal components, showed much better performance in terms of reproductive indicators than did the other breeds. Sows of all the breeds had short reproductive cycles, an indicator of intensified production, but also of appropriate herd management.

**Abstract:**

The present research aimed to study twelve reproductive indicators related to litter size and the farrowing interval for three maternal (Polish Large White, Polish Landrace, and Yorkshire) and three paternal (Duroc, Berkshire, Hampshire) breeds, raised on two farms in Poland and a farm in the United States. The study included 196 sows (45 Polish Large White, 37 Polish Landrace, 26 Berkshire, 33 Duroc, 40 Yorkshire, and 15 Hampshire), which altogether gave birth to 736 litters. The Kruskal-Wallis test was used to verify the influence of the breed on the reproductive traits, with a post-hoc procedure for pairwise comparisons implemented in the pgirmes of R. The adegenet, ade4, and factoextra packages of R were used to conduct multivariate analysis of the traits by means of principal component analysis. The breed significantly (*p* ≤ 0.05) influenced the following traits related to litter size: the total number of piglets born per litter, the number and percentage of piglets born alive per litter, the percentage of stillborn piglets per litter, the number and percentage of weaned piglets per litter; and those related to the farrowing interval: the lengths of gestation, lactation, the farrowing-to-conception interval, and the farrowing interval. The breed did not statistically significantly influence the number of stillborn piglets per litter and the length of the weaning-to-conception interval. Polish Landrace and Polish Large White sows had the highest numbers of born (for both, the mean of 14.0), born alive (12.9 and 12.7), and weaned piglets (11.5 and 10.5), which statistically significantly differed from these parameters in the other breeds. Polish Landrace sows significantly differed from all the other breeds in terms of the percentage of weaned piglets (84.1%), while Berkshire sows in terms of gestation length (118.4 days).

## 1. Introduction

The profitability of pig production strongly depends on the reproduction process, and sow reproductive performance results from features associated with litter size and farrowing interval [1]. The former include litter size (i.e., the total number of born piglets per litter), the number of born alive per litter, the number of stillborn per litter, and the number of weaned piglets per litter; the latter include the lengths of gestation, lactation, and weaning-to-conception interval, the last two periods combined creating the farrowing-to-conception interval.

Litter size and the number of piglets born alive constitute two crucial breeding performance indicators in pigs, both strongly affecting the profitability of pig production. Breeds considered maternal components—represented in Poland mainly by Polish Large White and Polish Landrace—have outstanding reproductive potential, strongly developed maternal instincts, and good milk production. Compared to maternal breeds, breeds considered paternal components—such as Duroc, popular in both Poland and the US, and Hampshire and Berkshire, popular in the US—achieve better fattening and slaughter performance, at the cost of inferior breeding performance [2]. 

Selection aimed at increasing litter size, however, increases piglet mortality [3] and shortens gestation [4]. Pointing to differences between breeds as well as between purebred individuals and crossbreds, some authors have suggested that the number of stillborn piglets per litter strongly depends on the breed [5,6]. 

The number of weaned piglets per litter depends on how many piglets were born alive in the litter, their birth weight, and the sow’s milk production [7]. The most important reason behind piglet mortality from birth to weaning is the low birth weight of piglets from numerous litters [8,9], because light piglets encounter various problems, such as with maintaining normal body temperature and struggling towards the udder and getting colostrum [10]. The percentage of weaned piglets, in turn, reflects the maternal instincts and care of the sow, thereby offering important information about breeding performance. Despite that, all too often this indicator is overlooked in studies on pig reproduction, as are the percentages of born alive and stillborn piglets. 

Gestation length in pigs ranges from 105 to 125 days, with a mean of 115 days [11]. It depends on various factors, among which the breed plays an important role, since differences in gestation length have been proved between different breeds [6,12] and between purebred individuals and crossbreds [13]. Gestation length also depends on litter size. In Sasaki and Koketsu’s [11] study, sows with the shortest gestation (up to 112 days) gave larger litters than females with the longest gestation (118 days and longer), but they also gave birth to more stillborn piglets. 

Mainly regulated by the breeder, lactation usually lasts from 14 to 28 days [14]. Recent years have witnessed the shortening of lactation in pigs, with farmers’ intention being to increase the frequency of farrows and, in that way, to maximize production. Such shortening, especially when overdone, does not come without cost, however. Lactation shorter than 14 days has been proved to negatively affect the subsequent growth of piglets and their welfare, but also the course of the next gestation of the sow [15]. 

Symptoms of oestrus in sows usually become visible between the third and seventh days after weaning [16], with differences in terms of the weaning-to-service interval length between breeds [17]. Purebred sows tend to have shorter weaning-to-service intervals than crossbred ones [18], as do multiparous sows compared to primiparous ones [19,20,21].

Lactation and the weaning-to-conception interval combine to create the farrowing-to-conception interval. Its optimal length is 36–63 days [19]. Too short an interval, particularly shorter than 28 days, will likely decrease the size of the next litter; too long can worsen the economic performance of breeding. This interval combined with gestation creates the farrowing interval. For most breeds, it ranges from 158 to 180 days, which translates to 1.8 to 2.2 farrows a year [22,23]. 

This study aimed to compare breeding performance indicators associated with litter size (litter size, the numbers of born alive, stillborn, and weaned piglets per litter, along with their per-litter percentages) and the farrowing interval (the lengths of gestation, lactation, the farrowing-to-conception interval, and the farrowing interval) in sows of breeds considered maternal and paternal components, kept on medium-sized farms in Poland and the USA.

## 2. Materials and Methods 

The study included 196 sows of six breeds: Polish Large White (45), Polish Landrace (37), Berkshire (26), Duroc (33), Yorkshire (40), and Hampshire (15). Among them, 23 were primiparous and 173 multiparous, with the share of primiparous sows ranging from 10 to 15% in all the breeds analyzed. Sows from different farms were not genetically related. Together, they gave birth to 736 litters, with 169 having been born in the spring, 194 in the summer, 167 in the autumn, and 206 in the winter. The sows included in the study were randomly chosen from among sows that did not have more than seven parities, without analyzing whole-life production. The mean number of parities for the breeds analyzed ranged from 2.13 (for Hampshire) to 5.51 (for Polish Large White). 

The animals were kept in three production farms of medium sizes, with the number of sows not exceeding 110; two were located in Poland and one in the United States, Texas (Table 1). In both farms in Poland, the sows were kept in closed piggeries with similar and standard microclimatic conditions; in the farm in the United States, the sows were kept in partially open buildings. In Texas, the cooling system (using sprinkling) was used to cool the sows when necessary. 

All the three farms used similar feeding and housing systems. From successful fertilization to the 90th day of pregnancy, sows were kept in group pens (housing a maximum of eight sows) and fed with complete feeding in the amount of 2.2–2.5 kg (about 11.5–12 MJ ME, 125 grams of crude protein, and 5 grams of lysine in 1 kg of feed). After the 90th day of gestation, the sows were placed in individual delivery pens and fed with complete feed in the amount of 2.8–3.0 kg (about 12.5 MJ ME, 170 grams of crude protein, and 8.5 grams lysine in 1 kg feed). All the sows had constant access to water. 

The three farms used only artificial insemination, and during oestrus, each sow was inseminated at least twice. None of the farms analyzed used chemical synchronization of oestrus, but each had a heat check boar. Piglets were usually weaned from the 26th and 30th days of life. Apart from exceptional situations, the farms applied neither litter equalization nor cross-fostering.

For each female, data on breeding performance were obtained for at least one full reproductive cycle, covering gestation, lactation, and the weaning-to-conception interval. From breeding documentation, information was collected on the dates of conception and weaning, litter size, and per-litter numbers of born alive, stillborn, and weaned piglets. For each litter, the percentages of born alive, stillborn, and weaned piglets were calculated as shares in the total number of piglets born in the litter.

For each sow, the following traits were also determined: gestation length, which is the number of days from conception to farrowing; lactation length, which is the number of days from farrowing to weaning; the length of weaning-to-conception interval, in days, meaning the period from weaning to a successful conception; the length of farrowing-to-conception interval, in days; and the length (in days) of farrowing interval, which covers the period from one effective conception to the next one.

This study was approved by the II Local Ethics Commission for Experiments Carried on Animals (Permit: No. 96/2015).

Statistical analysis was conducted using R 3.4.4 [24]. Summary statistics for the reproductive traits analyzed were determined using the pastecs package [25]. Each trait’s normality was verified using the Shapiro-Wilk test, and its variance homogeneity using Bartlett’s test. Since for all the traits the assumptions were violated, the data were analyzed using the Kruskal-Wallis non-parametric test, with the post-hoc procedure for pairwise comparisons implemented in the pgirmess package [26]. Principal component analysis (PCA) was applied using adegenet [27], ade4 [28] and factoextra [29] packages. Since the study aimed to determine the diversity of the reproductive indicators analyzed in sows kept in medium-sized farms, atypically short or long periods representing the various stages of reproductive cycle were not removed from the database.

## 3. Results

### 3.1. The Influence of the Breed on Sow Reproductive Indicators

Across the breeds, gestation lasted on average 115.2 days. A mean litter consisted of 12.2 piglets, of which 11.0 were born alive (90.0% of born alive), 1.2 (10.0%) were stillborn, and 9.1 (76.2%) were successfully weaned. Mean lactation lasted 27.1 days. Farrowing intervals lasted from 117 to 261 days, with the mean of 152.0 days. The mean weaning-to-conception interval took 10 days, and the mean farrowing-to-conception interval 37 days (Table 2).

The breed influenced almost all the traits, with two exceptions being the number of stillborn piglets per litter and the length of the weaning-to-conception interval (Table 3). Polish Landrace and Polish Large White sows had the largest litters (both 14.0), and Berkshire and Hampshire sows had the smallest (9.2 and 9.3, respectively). Not only did Polish Landrace and Polish Large White sows gave birth to the largest litters, but also to the most piglets born alive per litter (12.9 in Polish Landrace and 12.7 in Polish Large White sows). Berkshire (7.8) and Hampshire (8.0) sows gave birth to the fewest piglets born alive per litter. The percentage of born alive to all born did not vary too much between the breeds, from 86.3% in Yorkshire to 92.9% in Polish Landrace. The percentage of stillborn piglets showed greater variation, with only 6.6% in Polish Landrace and as high as 15.1% in Berkshire sows; Hampshire (13.5%) and Yorkshire (13.6%) litters had quite a high share of stillborn piglets. Polish Landrace and Polish Large White sows raised the largest litters, with the mean number of weaned piglets of 11.5 and 10.5, respectively. Hampshire, Berkshire and Yorkshire sows raised the smallest litters, with the mean number of weaned piglets being smaller than seven. In terms of the percentage of weaned piglets, Polish Landrace showed superiority over all the other breeds, with the mean of 84.0% of weaned piglets, while for the other breeds this indicator ranged from 69.5% for Yorkshire to 77.3% for Polish Large White. 

The longest gestation had Berkshire sows, with the mean of 118.4 days; the shortest, Duroc (114.6), Polish Large White (114.7) and Polish Landrace (114.9). The length of lactation ranged from 25.6 days for Berkshire to 27.8 days for Polish Landrace. Berkshire sows had the shortest farrowing-to-conception interval, with the mean of 35.3 days. Similar duration of this interval had Hampshire (the mean of 35.4 days) and Polish Large White (35.9) sows. The longest farrowing-to-conception interval was observed in Polish Landrace sows (40.6 days). Polish Landrace showed the longest farrowing interval, with the mean of 155.3 days.

### 3.2. Principal Component Analysis 

The first four principal components (PC1–PC4) explained nearly 90% of the variability in the data (Table 4). According to the Kaiser criterion, further analysis included only those components whose eigenvalues were at least one [30].

The first principal component, which explained 35% of the variation in the data, was strongly negatively correlated with the number and percentage of piglets born alive per litter and with the number of weaned piglets per litter, and strongly positively correlated with the percentage of stillborn piglets per litter (Table 5). The second principal component, explaining 25% of the variation in the data, was strongly negatively correlated with the farrowing and farrowing-to-conception intervals. The third principal component was strongly positively correlated with the total number of born piglets per litter, and the fourth, also positively, with lactation length.

**Figure 1:**
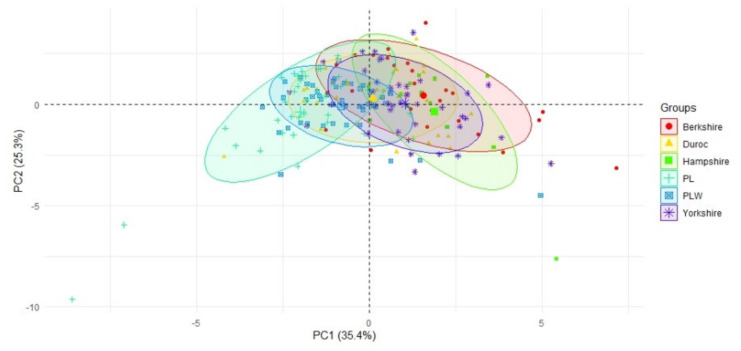
A scatterplot of the first principal component (PC) against the second PC, with the indication of the breeds. The first PC was correlated with the number and percentage of piglets born alive per litter and with the number of weaned piglets per litter (negative correlation), and the percentage of stillborn piglets per litter (positive correlation), while the second PC was correlated with the farrowing and farrowing-to-conception intervals (negative correlation). Axes represent loadings onto components 1 and 2.

**Figure 2:**
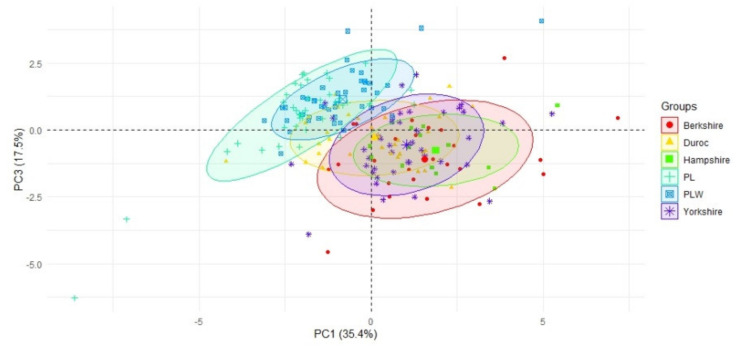
A scatterplot of the first PC against the third PC, with the indication of the breeds. The first PC was correlated with the number and percentage of piglets born alive per litter and with the number of weaned piglets per litter (negative correlation), and the percentage of stillborn piglets per litter (positive correlation), while the third PC was correlated with the total number of born piglets per litter (positive correlation). Axes represent loadings onto components 1 and 3.

**Figure 3:**
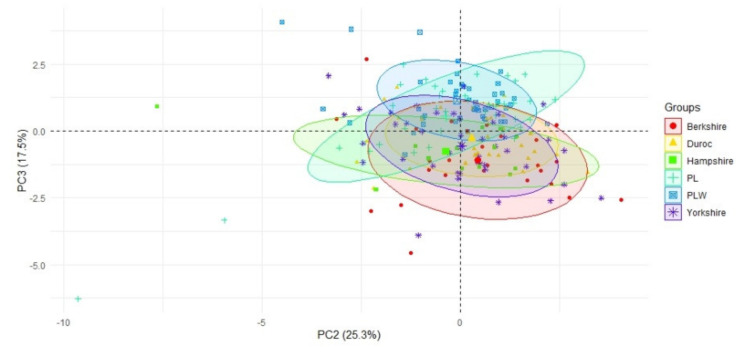
A scatterplot of the second PC against the third PC, with the indication of the breeds. The second PC was correlated with the farrowing and farrowing-to-conception intervals (negative correlation), while the third PC was correlated with the total number of born piglets per litter (positive correlation). Axes represent loadings onto components 2 and 3.

Figure 1, Figure 2 and Figure 3 indicate that Polish Large White and Polish Landrace sows gave birth to more alive piglets (in terms of both their number and percentage per litter) and had a smaller percentage of stillborn piglets than sows of the other breeds. Principal component analysis—PC1 and PC2 in particular—showed the greatest variation in the number of born alive and weaned piglets as well as in the percentage of weaned piglets between the sows of the Polish Landrace, Berkshire and Hampshire breeds (Figure 1). 

The analysis of PC1 against PC3 showed noticeable differences in the numbers of born, born alive, and weaned piglets per litter between the two Polish (Polish Large White and Polish Landrace) and two American (Hampshire and Berkshire) breeds. The greater the litter size, the more the piglets born alive and weaned per litter as well as the percentage of number born alive piglets per litter (Figure 2).

The analysis of PC2 (representing the lengths of the farrowing and farrowing-to-conception intervals) against PC3 (representing litter size) showed—as did the analysis of PC1 against PC2—that the greatest differences could be noticed between Polish Landrace and two American breeds, Berkshire and Hampshire (Figure 3). Polish Large White and Polish Landrace sows had the largest litters, but the former had more unified lengths of the farrowing and farrowing-to-conception intervals.

## 4. Discussion

The differences in litter size between the breeds have several reasons. First, they were bred for different traits. The maternal breeds (Polish Large White, Polish Landrace, and Yorkshire) were bred mainly to improve reproductive traits, while the paternal ones (Duroc, Hampshire, and Berkshire) to improve production traits. Breeding to improve both production and reproductive traits would unlikely succeed, because they are either negatively or weakly positively correlated [31,32,33], which translates into lower reproductive indicators in breeds perfected for meatiness. Given the above knowledge, however, we decided to compare six breeds that are considered either the maternal or the paternal component and that are popular in medium-sized farms, in which the number of sows creating the basic herd is close to 100. Contemporary trends aiming at improving animal welfare, along with the increasing negative perception of industrial methods used in breeding, incline to study whether the reproduction of pigs on medium-farms can be as effective as on industrial farms.

We observed the largest litters in Polish Large White and Polish Landrace (both 14.0) sows. The other breeds had significantly smaller litters, the smallest ones having been observed in Berkshire (9.2) and Hampshire (9.3) sows. These data confirm previous results. Studying breeding performance indices of Hampshire sows kept in breeding herds in Sweden, Tummaruk et al. [34] observed the mean litter size of 9.8 piglets, a higher value than the 7.8 reported by Rico [35], and similar to the 9.9 reported by Bass et al. [36]. In our studies, Yorkshire sows gave birth to litters with the mean of 10.2 piglets. Tantasuparuk et al. [22] reported lower (9.1) while Tummaruk et al. [17] higher (11.5) values. Litter sizes we observed in Polish Large White and Polish Landrace sows were close to those reported by other authors. Studying Polish Large White sows, Milewska [23] observed the mean of 11.8 and Schwarz et al. [37] the mean of 11.6 piglets born per litter. Knecht and Duziński [21] reported a smaller value (10.4). 

Sasaki and Koketsu [11] showed—and Chen et al. [13] confirmed—that the larger the litter size, the shorter the gestation length, and the more stillborn piglets per litter. Sows whose gestation lasted shorter than 113 days gave birth to litters with the mean of 2.3 stillborn piglets; those whose gestation lasted at least 118 days, the mean of only 1.1; and those whose gestation lasted 115–116 days, the mean of one stillborn piglet per litter. In Imboonta and Kuhaaudomlarp’s [6] study, both short (≤114 days) and long (≥120 days) gestation increased the number of stillborn piglets per litter (the means of 0.98 and 0.95, respectively), and sows with gestation lasting for 117 days gave birth to litters with the fewest stillborn piglets (0.71). Our results were similar, in that the mean number of stillborn piglets ranged from 0.95 in Duroc to 1.40 in Yorkshire sows. Most authors, however, have reported litters with slightly fewer stillborn piglets. In Milewska’s [23] research, Polish Large White sows gave birth to litters with the mean of 0.56 stillborn piglets per litter, which was less than the 0.91 reported by Schwarz et al. [37]. Studying reproductive performance indices of six maternal lines during the first four farrowings, Moeller et al. [38] observed the mean of 0.81 stillborn piglets per litter.

The mean numbers of stillborn piglets we observed for the six breeds did not statistically significantly differ between the breeds. This may indicate that all of them reached optimal litter sizes and all the farrowings were successful, since the mortality of piglets increases in both most and least numerous litters. Herpin et al. [39] found that as the litter size increases, the risk of prolonging the duration of the farrowing increases, thereby increasing the risk of hypoxia and the resulting in death of the piglet, especially in the last-born one. Piglets from the smallest litters, in turn, may be too heavy, which may hinder and prolong the farrowing [40].

The percentage of stillborn piglets usually ranges from 2 to 15% [22,39,41]. In our study, it ranged from 6.8% in Polish Landrace to 15.1% in Berkshire sows. We found statistically significant differences between Polish Landrace and Berkshire, Hamspshire and Yorkshire breeds. These differences might have been primarily due to large differences in terms of the total number of born piglets per litter, not of the number of stillborn piglets per litter. Since the percentage of stillborn piglets in a litter is determined as their number to litter size, more piglets per litter along with a relatively unchanging number of stillborn piglets will lead to a smaller percentage of stillborn piglets per litter in the most fertile sows. 

We also found great differences between the breeds in terms of the number of piglets born alive. Most alive piglets were born in the litters of Polish Landrace (12.9) and Polish Large White (12.7) sows, and least in the litters of Berkshire (7.8), Hampshire (8.0) and Yorkshire (8.9) sows. The results for the Polish Landrace and Polish Large White breeds were close to those reported by Blicharski et al. [42], who observed the mean of 12.2 of piglets born alive to Polish Landrace sows and 12.3 to Polish Large White ones. Analyzing litter size in sows from the first to the sixth farrowing in these two breeds, Szostak and Katsarov [1] reported the mean numbers of piglets born alive per litter ranging from 11.5 to 13.3 in Polish Landrace sows and from 11.6 to 12.4 in Polish Large White ones. Knecht and Duziński [21], however, reported smaller values (10.5). In our study, Yorkshire sows gave birth to litters with the mean of 8.8 piglets born alive, a result close to that reported by Tantasuparuk et al. [22]. In a long-term study of reproductive indicators, conducted for 1984–1999, Chen et al. [43] observed the mean of 10.6 piglets born alive per litter to Yorkshire sows. The mean numbers of piglets born alive to Duroc and Hampshire sows were much smaller than the 11.5 and 11.9 reported by Blicharski et al. [42], respectively. McMullen [44] observed that Berkshire sows gave birth to litters with the mean of nine alive piglets, a higher number than the 7.8 we observed.

The above-discussed variation in the number of piglets born alive per litter largely resulted from variation in litter size and the number of stillborn piglets per litter. Above, we have discussed factors affecting these two traits, and the same factors influence the number and percentage of piglets born alive per litter. This percentage was the highest in sows of the Polish Landrace (92.9%) and Polish Large White (91.44%) breeds, and the lowest in Berkshire sows (84.9%). We can apply here a similar interpretation to that we applied for the percentage of stillborn piglets per litter: Since the percentage of piglets born alive in a litter is determined as their number to all piglets born in the litter, more piglets in litters with a relatively equal number of stillborn piglets will mean a higher percentage of piglets that were born alive to the most fertile sows.

Postnatal survival can be smaller in larger litters, because such piglets usually have a lower birth weight [45,46]. It makes it difficult for them to struggle towards the udder and collect an adequate amount of colostrum, a source of nutrients and antibodies providing the necessary immune protection [46]. Light piglets are also more likely to be crushed by the sow or to lose body temperature [10].

We did not observe, however, an increase in piglet mortality in sows with the largest litters. The sows of Polish Large White and Polish Landrace, which had the largest litters, both in terms of all born piglets and those alive, raised the most piglets, with the means of 10.5 and 11.5 weaned piglets, respectively. Blicharski et al. [42] reported similar values (11.3 for Polish Large White and 11.4 for Polish Landrace), and so did Milewska [23]. Schwarz et al. [37], however, reported smaller values of this parameter for the Polish Large White breed, that is, the mean of 8.2 weaned piglets per litter. Considering the reasons behind so much smaller a value of this indicator, we should remember that sows kept in the conditions of large-scale production, as was the case in Schwarz et al. [37] study, seldom get adequate attention, also during the rearing of piglets. Milewska’s [23] results confirm this reasoning: She showed that the larger the herd, the smaller the number of weaned piglets per litter. Polish Large White sows are more aggressive and nervous than Polish Landrace ones, and more often have problems with milk production due to the mastitis, metritis and agalactia (MMA) syndrome, three possible reasons behind the increased mortality of piglets during rearing [37]. The mean numbers of weaned piglets per litter we observed in these two breeds may indicate good animal care as well as appropriate herd management, technological solutions used, and microclimate. 

The percentage of weaned piglets per litter ranged from 69.5% in Yorkshire to 84.1% in Polish Landrace sows. The latter breed statistically significantly differed in terms of this indicator from the other breeds, showing its superiority. Berkshire and Hampshire sows, both having the smallest litters in terms of litter size and the number of piglets born alive, had similar percentages of weaned piglets (71.9 and 70.0%, respectively). Polish Large White and Duroc, although quite different in terms of litter size, had similar percentages of weaned piglets per litter: 77.3% and 75.7%. This indicates a slightly increased number of falls of piglets during rearing in Polish Large White sows, a result in line with Schwarz et al. [37] observations.

The number of weaned piglets by a sow during a year depends not only on factors related to litter size, but also on those related to the length of the reproductive cycle. Depending on the duration of gestation, lactation, and the weaning-to-conception interval, the length of the farrowing interval affects farrowing frequency, that is, the number of litters obtained from the sow during a calendar year.

Mean gestation length in our study was 115.2 days, which corresponds to the normal physiological length of gestation in pigs. It is also similar to the results reported by other authors, who reported mean gestation length ranging from 112.2 to 117.0 days [6,45,47,48]. In our study, Duroc, Polish Landrace, and Polish Large White sows had the shortest gestation while Berkshire ones had the longest, with the mean of 118.4 days. This observation confirms McMullen’s [44], who indicated that Berkshire sows have longer gestation, ranging from 116 to 118 days, than have other breeds.

Gestation length in sows depends on both genetic and environmental factors. This parameter has been proved to differ between pure breeds [49,50] as well as between crossbreeds [6,11]. Environmental factors influencing gestation length may include the time of year, sow age, and closely related the number of farrows of the sow [37]. Most studies, however, have shown that gestation length strongly correlated to litter size, a relationship likely related to the limited uterine capacity [51,52]. Sows with the largest litters usually have the shortest gestation. In Sasaki and Koketsu’s [11] study, sows with gestation longer than 118 days had smaller litters than those with gestation ranging from 112 to 117 days. Imboonta and Kuhaaudomlarp [6] showed that sows whose gestation lasted below 114 days gave birth to the mean of 11.4 piglets per litter, while those whose gestation lasted over 120 days had litter size smaller by over one piglet. Similar results were obtained by Chen et al. [13], who stated that the shorter the gestation, the larger the litter. We also observed such a relationship: Polish Large White and Polish Landrace sows, which gave birth to the largest litters, had gestation statistically significantly shorter than did Berkshire and Yorkshire sows, both having smaller litters.

These days, lactation in pigs usually lasts for 28 days, a consequence of both legal regulations and care for animal welfare [53]. The mean length of lactation of all the sows in our study was 27.2 days, a similar result to that by Ziedina et al. [54]. However, being the grand mean, this estimate ignores the between-breed differences, and we did observe them. Polish Landrace sows, who gave the largest numerous litters, had also the longest lactation (the mean of 27.8 days), while Berkshire sows, who gave the smallest litters, had the shortest lactation (the mean of 25.6 days, being statistically significantly smaller than that for Polish Landrace).

It is possible that the farmers used longer lactation in the most fertile sows not only because sows giving birth to the largest litters have the shortest gestation, but also because more piglets have to share the amount of milk their mothers produce. In large litters, the piglets have to struggle for milk, with stronger ones collecting more of it than the weaker ones. On average, however, piglets from large litters are likely to have smaller mean daily growth and, in turn, smaller mean body weight than those from small litters, as they are not so much affected by the need to struggle to feed. It is body weight that is often a key factor behind the decision to wean or to wait. This phenomenon is observed especially in the summer, since owing to high ambient temperatures, sows eat less and thus produce less milk to be shared by their piglets, and so their body weight decreases [19].

Some authors, however, have reported much shorter lactation lengths, like 17.4 [55] and 15.0 [38]. Both these studies dealt with pig production in the United States, so one might conclude that such drastic shortening of lactation may have resulted from using the so-called segregated early weaning, a production system aiming to increase production efficiency by weaning piglets at the age of 10–20 days, popular in the United States [55,56]. Our study did not support this thesis: The mean length of lactation for sows kept in Texas ranged from 25.6 in Berkshire to 27.7 days in Duroc sows. These results do not come as a surprise, however: Knauer and Hostetler [57] showed that the United States had been experiencing a trend of increasing lactation length in sows. 

Shortening lactation, especially below 14 days, does have negative consequences [14,15,58], possibly lengthening the non-production period [56]. Kokestu and Dial [19] showed that the mean length of the non-productive period in sows with lactation lasting from 1 to 7 days was 22.3 days, while that in sows with lactation lasting over four weeks was 5.5 days. So drastic an effect of lactation length on the length of the non-productive period may result from a disturbed hormonal balance or more frequent embryo resorption in sows mated too early, possibly due to the incomplete involution of the uterus [53]. Our study showed something else, however: The longest non-productive period occurred in the sows with the longest lactation, and these sows also raised the largest litters. According to Koketsu et al. [56], sows with prolonged lactation are more likely to lose body weight and use more energy reserves, which can in turn prolong the non-productive period. The maximum length of the weaning-to-conception interval in Polish Landrace sows was 118 days, while in the Hampshire and Berkshire breeds 27 and 53 days, respectively. So long weaning-to-conception intervals would unlikely stay neutral to the non-productive period, rather increasing its duration in the Polish Landrace breed.

Lactation and the weaning-to-conception interval combine into the farrowing-to-conception interval. In our study, its mean length ranged from 35.3 days in Berkshire to 40.6 days in Polish Landrace sows. The optimal length of the farrowing-to-conception interval is about 50 days [55]. We failed to find out related research with which we could check the lengths of the interval from farrowing to conception in the breeds studied. A possible reason is that most authors analyze the weaning-to-first-service interval, but the first service does not always succeed, and so not always does it end in gestation. Sows with both very short and very long lactation can have problematic conception, despite showing the first symptoms of oestrus several days after weaning the piglets. This would result from problems with embryo implantation, which could be due to great weight loss in sows with very long lactation [59] or incomplete uterine involution in sows with very short lactation [58]. For this very reason, the weaning-to-conception interval may be more informative than is the weaning-to-first-service interval.

Even the longest weaning-to-conception interval we observed—which was for the Polish Landrace breed—was noticeably shorter than the optimal length of this interval, which is around 50 days. So short farrowing-to-conception intervals may mean that the three farms were managed under intensive production systems. On the other hand, five or six weeks from farrowing to conception should be enough for a sow’s reproductive system to fully regenerate and for the sow to return to its reproductive ability [60].

Shortening lactation and the non-productive period shortens the farrowing interval, thereby increasing the number of farrows. Across all the breeds, the mean length of the farrowing interval was 152.0 days. Although the breeds statistically significantly differed in terms of this parameter, the differences were rather minor, with the farrowing interval lengths ranging from 150.5 in Hampshire to 155.3 days in Polish Landrace sows. Blicharski et al. [42] reported longer intervals, but Schwarz et al. [37] observed similar values (152.8), as did Knecht and Duziński [21], in whose study the farrowing interval ranged from 153 to 160 days, depending on the month. Yorkshire sows in Sweden [17] and Thailand [22] had longer reproductive cycles (168.3 and 160.5 days, respectively). Nagyne-Kiszlinger et al. [50] presented similar results for white breeds reared in Hungary (Hungarian Large White and Hungarian Landrace), but Milewska [23] reported longer reproductive cycles in Polish Large White sows, on average lasting 180 days.

The above-discussed results offer a similar picture to that drawn by principal component analysis, which also showed that Polish Landrace and Polish Large White sows gave birth to larger litters, in terms of litter size (PC1 and PC3) and the number and percentage of piglets born alive (PC1 and PC2) than did sows of other breeds.

Other factors likely affecting reproductive performance in sows include herd management, work organization, and feeding. The three farms studied had similar herd management and similar numbers of sows in the basic herd and used similar feeding (with a very even composition of mixed feed). Despite these similar conditions, however, we did observe some differences between them, in terms of reproductive performance—an expected result, since in production conditions, it is impossible to avoid the effects of conditions (however similar they can seem) and animal welfare, not to mention between-individual variation, typical of all biological populations. For this very reason, we included three farms, thereby adding this variation into the results. However, analyzing the between-farm variation itself did not constitute our aim here. Since we studied only three farms, the farm effect would have to be considered as fixed. Thus, this effect would have to be interpreted only in relation to these very farms, without any possibility to generalize the results. In order to analyze a true farm effect, one would need to design a different study, with a large number of farms selected in a random manner, so that the farm effect could be analyzed as random, and as such could be generalized to the population of farms.

Although we did not analyze the seasonal effect, we need to remember that this factor might affect litter size, decreasing reproductive performance in the summer [61,62], especially in the farm located in Texas. Given the climate in Texas, this factor could in some way affect breeding performance, and maybe even deepen the differences between the breeds. On the other hand, the farm used a cooling system, which in some way neutralized the effects of high temperatures. Additionally, the research included medium-scaled pig farms, which are known to offer better welfare for animals than industrial farms. 

The research allowed us to broaden our knowledge on the diversity of reproductive performance indicators of the most popular pig breeds pigs kept in Poland (Polish Large White and Polish Landrace) and the United States (Duroc, Hampshire, Berkshire, and Yorkshire). In doing so, we analyzed reproductive indicators associated with litter size and the farrowing interval in sows of breeds considered either maternal or paternal components kept on medium-sized farms, an approach that enabled us to take a broad view on pig reproduction.

## 5. Conclusions

In the study, Polish Large White and Polish Landrace sows, both considered maternal components, showed better reproductive performance related to litter size than did the other breeds: These sows gave birth to the mean of 14 piglets per litter, while sows of the other breeds from about nine (Berkshire and Hampshire) to 10.5 (Duroc, Yorkshire) piglets per litter. Polish Landrace and Polish Large White sows, with almost 13 alive piglets per litter, were also superior to the other breeds in terms of the mean number of piglets born alive, which ranged from 7.8 in Berkshire to 9.5 in Duroc sows. Sows of the Hampshire, Berkshire and Yorkshire breeds weaned, on average, fewer than seven piglets per litter; both Polish Large White (the mean of 10.5 weaned piglets) and Polish Landrance (11.5) sows performed better. On the other hand, the breeds did not significantly differ in terms of the number of stillborn piglets, a result suggesting optimal litter sizes as well as successful farrowings in all the breeds. The mean length of the reproductive cycle ranging from 150.5 (in Hampshire sows) to 155.3 (in Polish Landrace sows) days testifies to the maximization of production and the minimization of the non-productive period’s length (its mean ranging from almost nine days in Polish Large White and Hampshire sows to 12.7 days in Polish landrace sows) on the three farms, indicating proper work organization and herd management.

## Figures and Tables

**Table 1:** The number of sows studied per breed.

Farm	The Farm’s Location	Breed	Number of Sows	Sum
I	Poland	Polish Large White	15	56
		Polish Landrace	27	
		Duroc	14	
II	Poland	Polish Large White	30	40
		Polish Landrace	10	
III	USA	Duroc	19	100
		Hampshire	15	
		Yorkshire	40	
		Berkshire	26	

**Table 2:** Summary statistics for the analyzed reproductive traits of sows (across the breeds studied).

Trait	Mean	Median	SD	Min	Max	CV (%)
Gestation length (days)	115.2	115.0	2.01	104	125	1.7
Total no. of born piglets	12.2	12.0	3.56	2	23	29.2
Number of piglets born alive	11.0	11.0	3.39	0	20	30.9
Percentage of piglets born alive	90.0	92.9	0.13	0	100	15.0
Number of stillborn piglets	1.23	1.00	1.61	0	13	132.6
Percentage of stillborn piglets	9.96	7.14	0.13	0	100	133.5
Number of weaned piglets	9.12	10.00	2.92	0	16	32.0
Percentage of weaned piglets	76.2	78.6	0.18	18	100	24.3
Lactation length (days)	27.2	27.0	3.28	0	45	12.1
Weaning-to-conception interval (days)	9.9	5.0	12.65	2	118	127.7
Farrowing-to-conception interval (days)	37.1	37.0	13.20	2	145	8.7
Farrowing interval (days)	152.0	148.0	13.01	117	261	35.1

SD: standard deviation; CV: coefficient of variation.

**Table 3:** Summary statistics for the analyzed reproductive traits of sows of the breeds studied.

Trait		Breed					
		PL	PLW	Duroc	Berkshire	Hampshire	Yorkshire
Number of litters		150	258	99	58	32	139
Gestation length (days)	mean	114.7 ^c^	114.9 ^c^	114.6 ^c^	118.4 ^a^	115.1 ^b,c^	115.4 ^b^
	median	115.0	115.0	115.0	119.0	115.0	115.0
	SD	1.64	1.41	2.15	2.54	1.24	1.86
	CV (%)	1.4	1.2	1.9	1.9	1.1	1.6
Total no. of born piglets	mean	14.00 ^a^	14.00 ^a^	10.39 ^b^	9.16 ^b^	9.25 ^b^	10.17 ^b^
	median	14.0	14.0	11.0	9.0	9.5	11.0
	SD	2.70	3.13	2.77	2.49	2.41	3.19
	CV (%)	19.4	22.4	26.7	27.2	26.1	31.4
No. of piglets born alive	mean	12.93 ^a^	12.67 ^a^	9.46 ^b^	7.81^c^	8.00 ^b,c^	8.75 ^b,c^
	median	13.00	13.00	10.00	8.00	8.00	9.00
	SD	2.50	2.80	2.64	2.55	2.53	2.89
	CV (%)	19.3	22.1	27.9	32.7	31.6	33.1
Percentage of piglets born alive	mean	92.9 ^a^	91.4 ^a,b^	91.2^a, b^	84.9 ^b^	86.5 ^b^	86.3 ^b^
	median	100.0	93.8	100.0	90.0	88.9	90.9
	SD	0.12	0.11	0.12	0.19	0.13	0.15
	CV (%)	13.0	12.4	13.2	22.1	15.3	18.0
No. of stillborn piglets	mean	1.01	1.34	0.95	1.34	1.25	1.40
	median	0.00	1.00	0.50	1.00	1.00	1.00
	SD	1.46	1.91	1.23	1.47	1.41	1.57
	CV (%)	144.6	142.1	129.9	109.3	113.1	112.1
Percentage of stillborn piglets	mean	6.57 ^b^	8.80 ^a,b^	8.92 ^a,b^	15.09 ^a^	13.46 ^a^	13.55 ^a^
	median	0.00	6.25	3.12	10.00	11.11	9.09
	SD	0.09	0.12	0.12	0.18	0.13	0.15
	CV (%)	141.6	138.8	134.5	124.3	98.3	114.4
No. of weaned piglets	mean	11.5 ^a^	10.5 ^b^	7.8 ^c^	6.5 ^d^	6.2 ^d^	6.8 ^d^
	median	12.0	10.0	8.0	6.0	6.0	7.0
	SD	2.06	1.87	2.68	2.29	1.60	2.33
	CV (%)	17.9	17.9	34.4	35.2	25.8	34.3
Percentage of weaned piglets	mean	84.1 ^a^	77.3 ^b^	75.7 ^b^	71.9 ^b^	70.0 ^b^	69.5 ^b^
	median	85.2	77.8	80.0	75.0	73.9	71.4
	SD	0.14	0.16	0.20	0.22	0.18	0.21
	CV (%)	16.6	20.4	27.1	30.2	25.9	30.8
Lactation length (days)	mean	27.8 ^a^	27.1 ^a,b^	27.7 ^a,b^	25.6 ^c^	26.5 ^b,c^	27.1 ^b^
	median	28.0	27.0	28.0	26.0	26.0	27.0
	SD	4.12	2.27	3.53	3.29	2.28	3.63
	CV (%)	14.9	8.4	12.8	12.9	8.6	13.4
Weaning-to-conception interval (days)	mean	12.7	8.8	9.4	9.8	8.9	9.6
	median	5.0	5.0	5.5	5.0	6.0	5.0
	SD	19.43	9.61	10.55	10.39	6.96	11.41
	CV (%)	152.6	109.2	112.0	106.6	78.5	118.4
Farrowing-to-conception interval (days)	mean	40.6 ^a^	35.9 ^a,b^	37.4 ^a,b^	35.3 ^b^	35.4 ^a,b^	36.4 ^a,b^
	median	34.0	33.0	34.0	32.0	32.0	33.0
	SD	18.92	9.63	11.69	10.48	7.39	12.75
	CV (%)	46.6	26.8	31.2	29.7	20.9	35.0
Farrowing Interval (days)	mean	155.3 ^a,b^	150.6 ^b^	152.0 ^a,b^	153.7 ^a^	150.5 ^a,b^	151.8 ^a,b^
	median	148.0	147.0	149.0	149.0	148.0	148.0
	SD	19.25	9.92	11.72	11.21	7.05	13.01
	CV (%)	12.4	6.6	7.7	7.3	4.7	8.6

^a–d^ different letters indicate statistically different means at *p* < 0.05; SD: standard deviation; CV: coefficient of variation; PL: Polish Landrace; PLW: Polish Large White.

**Table 4:** Eigenvalues, share in total variation and cumulative share in total in the data of the first four principal components.

	PC1	PC2	PC3	PC4
Eigenvalue	4.25	3.04	2.10	1.19
Variation (%)	35.4	25.3	17.5	9.9
Cumulated variation (%)	35.4	60.7	78.2	88.2

PC: principal component.

**Table 5:** Correlation coefficients between the original traits and the first four principal components.

Trait	Principal Component			
	PC1	PC2	PC3	PC4
Gestation length (days)	0.28	0.07	−0.35	−0.64
Total number of born piglets	−0.55	−0.35	0.72	−0.17
Number of piglets born alive	−0.79	−0.08	0.55	−0.12
Percentage of piglets born alive	−0.74	0.60	−0.19	0.09
Number of stillborn piglets	0.56	−0.66	0.42	−0.12
Percentage of stillborn piglets	0.74	−0.60	0.18	−0.09
Number of weaned piglets	−0.79	−0.11	0.47	−0.25
Percentage of weaned piglets	−0.61	0.40	−0.18	−0.18
Lactation length (days)	−0.22	−0.30	0.11	0.76
Weaning-to-conception interval (days)	−0.50	−0.68	−0.48	−0.11
Farrowing interval (days)	−0.49	−0.71	−0.49	−0.06
Farrowing-to-conception interval (days)	−0.54	−0.72	−0.42	0.07

## References

[1] Szostak B., Katsarov V. (2013). Reproductive performance of Polish Large White and Polish Landrace sows. Agric. Sci. Technol..

[2] Knecht D., Jankowska-Mąkosa A., Środoń S. (2013). Najważniejsze cechy użytkowości rozrodczej świń. Hodowca Trzody Chlewnej.

[3] Johnson R.K., Nielsen M.K., Casey D.S. (1999). Responses in ovulation rate, embryonal survival, and litter traits in swine to 14 generations of selection to increase litter size. J. Anim. Sci..

[4] Hanenberg E.H.A.T., Knol E.F., Merks J.W.M. (2001). Estimates of genetic parameters for reproduction traits at different parities in Dutch Landrace pigs. Livest. Prod. Sci..

[5] Bidanel J.P. (1993). Estimation of crossbreeding parameters between Large White and Meishan porcine breeds. III. Dominance and epistatic components of heterosis on reproductive traits. Genet. Sel. Evol..

[6] Imboonta N., Kuhaaudomlarp P. (2012). Genetic associations between stillbirth, total number of piglets born and gestation length in a commercial pig farm. Thai J. Vet. Med..

[7] Lay D.C., Matteri R.L., Carroll J.A., Fangman T.J., Safranski T.J. (2002). Pre-weaning survival in swine. J. Anim. Sci..

[8] Knol E.F., Leenhouwers J.I., Van der Lende T. (2002). Genetic aspects of piglet survival. Livest. Prod. Sci..

[9] Canario L., Cantoni E., Le Bihan E., Caritez J.C., Billon Y., Bidanel J.P., Foulley J.L. (2006). Between-breed variability of stillbirth and its relationship with sow and piglet characteristics. J. Anim. Sci..

[10] Panzardi A., Bernardi M.L., Mellagi A.P., Bierhals T., Bortolozzo F.P., Wentz I. (2013). Newborn piglet traits associated with survival and growth performance until weaning. Prev. Vet. Med..

[11] Sasaki Y., Koketsu Y. (2007). Variability and repeatability in gestation length related to litter size in female pigs on commercial farms. Theriogenology.

[12] Wilkie P.J., Paszek A.A., Beattie C.W., Alexander L.J., Wheeler M.B., Schook L.B. (1999). A genomic scan of porcine reproductive traits reveals possible quantitative trait loci (QTLs) for number of corpora lutea. Mamm. Genome..

[13] Chen C.Y., Guo Y.M., Zhang Z.Y., Ren J., Huang L.S. (2010). A whole genome scan to detect quantitative trait loci for gestation length and sow maternal ability related traits in a White Duroc × Erhualian F2 resource population. Animal.

[14] Weaver A.C., Kind K.L., Terry R., van Wettere W.H. (2014). Effects of lactation length and boar contact in early lactation on expression of oestrus in multiparous sows. Anim. Reprod. Sci..

[15] Gerritsen R., Soede N.M., Langendijk P., Taverne M.A.M., Kemp B. (2008). Early embryo survival and development in sows with lactational ovulation. Reprod. Domest. Anim..

[16] Kemp B., Soede N.M., Langendijk P. (2005). Effects of boar contact and housing conditions on estrus expression in sows. Theriogenology.

[17] Tummaruk P., Lundeheim N., Einarsson S., Dalin A.M. (2000). Reproductive performance of purebred Swedish Landrace and Swedish Yorkshire Sows: II. Effect of mating type, weaning-to-first-service interval and lactation length. Acta Agric. Scand. A Anim. Sci..

[18] Suwanasopee T., Mabry J.W., Koonawootrittriron S., Sopannarath P., Tumwasorn S. (2005). Estimated genetic parameters of non-productive sow days related to litter size in swine raised in Thailand. Thai J. Agric. Sci..

[19] Koketsu Y., Dial G.D. (1997). Factors influencing the postweaning reproductive performance of sows on commercial farms. Theriogenology.

[20] Tummaruk P., Tantasuparuk W., Techakumphu M., Kunavongkrit A. (2010). Influence of repeat-service and weaning-to-first-service interval on farrowing proportion of gilts and sows. Prev. Vet. Med..

[21] Knecht D., Duziński K. (2014). The effect of parity and date of service on the reproductive performance of Polish Large White × Polish Landrace (PLW × PL) crossbred sows. Ann. Anim. Sci..

[22] Tantasuparuk W., Lundeheim N., Dalin A.M., Kunavongkrit A., Einarsson S. (2000). Reproductive performance of purebred Landrace and Yorkshire sows in Thailand with special reference to seasonal influence and parity number. Theriogenology.

[23] Milewska W. (2006). Production traits of Polish Large White sows kept in breeding herds in the Warmia and Mazury region in the years 1998–2002. Anim. Sci. Pap. Rep..

[24] R Core Team (2018). R: A Language and Environment for Statistical Computing.

[25] Grosjean P., Ibanez F. (2018). Pastecs: Package for Analysis of Space-Time Ecological Series. R Package Version 1.3.21. http://CRAN.R-project.org/package=pastecs.

[26] Giraudoux P. (2018). Pgirmess: Spatial Analysis and Data Mining for Field Ecologists. R Package Version 1.6.9. https://CRAN.R-project.org/package=pgirmess.

[27] Jombart T., Ahmed I. (2011). Adegenet 1.3-1: New tools for the analysis of genome-wide SNP data. Bioinformatics..

[28] Bougeard S., Dray S. (2018). Supervised multiblock analysis in R with the ade4 package. J. Stat. Softw..

[29] Kassambara A., Mundt F. (2017). Factoextra: Extract and Visualize the Results of Multivariate Data Analyses. R Package Version 1.0.5. https://CRAN.R-project.org/package=factoextra.

[30] Kaiser H.F. (1960). The application of electronic computers to factor analysis. Educ. Psychol. Meas..

[31] Hermesch S., Luxford B.G., Graser H.U. (2000). Genetic parameters for lean meat yield, meat quality, reproduction and feed efficiency traits for Australian pigs 3. Genetic parameters for reproduction traits and genetic correlations with production, carcass and meat quality traits. Livest. Prod. Sci..

[32] Holm B., Bakken M., Klementsdal C., Vangen O. (2004). Genetic correlations between reproduction and production traits in swine. J. Anim. Sci..

[33] Lee J.H., Song K.D., Lee H.K., Cho K.H., Park H.C., Park K.D. (2015). Genetic parameters of reproductive and meat quality traits in Korean Berkshire pigs. Asian Australas. J. Anim. Sci..

[34] Tummaruk P., Lundeheim N., Einarsson S., Dalin A.M. (2001). Reproductive performance of purebred Hampshire sows in Sweden. Livest. Prod. Sci..

[35] Rico C. (1988). Reproductive performance of the Hampshire breed. Cuba. J. Agric. Sci..

[36] Baas T.J., Christian L.L., Rothschild M.F. (1992). Heterosis and recombination effects in Hampshire and Landrace swine: I. Maternal traits. J. Anim. Sci..

[37] Schwarz T., Nowicki J., Tuz R. (2009). Reproductive performance of Polish Large White sows in intensive production—Effect of parity and season. Ann. Anim. Sci..

[38] Moeller J.S., Goodwin R.N., Johnson R.K., Mabry J.W., Baas T.J., Robison O.W. (2004). The National Pork Producers Council Maternal Line National Genetic Evaluation Program: A comparison of six maternal genetic lines for female productivity measures over four parities. J. Anim. Sci..

[39] Herpin P., Hulin J.C., Le Dividich J., Fillaut M. (2001). Effect of oxygen inhalation at birth on the reduction of early postnatal mortality in pigs. J. Anim. Sci..

[40] Dziuk P. (1979). Control and mechanics of parturition in the pig. Anim. Reprod. Sci..

[41] Oliviero C., Heinonen M., Valros A., Halli O., Peltoniemi O.A. (2008). Effect of the environment on the physiology of the sow during late pregnancy, farrowing and early lactation. Anim. Reprod. Sci..

[42] Blicharski T., Polok P., Snopkiewicz M. (2018). Wyniki oceny trzody chlewnej w 2017 roku.

[43] Chen P., Baas T.J., Mabry J.W., Koehler K.J., Dekkers J.C.M. (2003). Genetic parameters and trends for litter traits in U.S. Yorkshire, Duroc, Hampshire, and Landrace pigs. J. Anim. Sci..

[44] McMullen L.K. (2006). Berkshire Niche Market Opportunity Guidelines (PN03-05B).

[45] Leenhouwers J.I., van der Lende T., Knol E.F. (1999). Analysis of stillbirth in different lines of pig. Livest. Prod. Sci..

[46] Baxter E.M., Jarvis S., D’Eath R.B., Ross D.W., Robson S.K., Farish M., Nevison I.M., Lawrence A.B., Edwards S.A. (2008). Investigating the behavioural and physiological indicators of neonatal survival in pigs. Theriogenology.

[47] Cassady J.P., Young L.D., Leymaster K.A. (2002). Heterosis and recombination effects on pig reproductive traits. J. Anim. Sci..

[48] Casellas J., Varona L., Munoz G., Ramirez O., Barragan C., Tomas A., Martinez-Giner M., Ovilo C., Sanchez A., Noguera J.L. (2008). Empirical Bayes factor analyses of quantitative trait loci for gestation length in Iberian x Meishan F2 sows. Animal.

[49] Kennedy B.W., Moxley J.E. (1978). Genetic and environmental factors influencing litter size, sex ratio, and gestation length in the pig. Anim. Sci. J..

[50] Nagyne-Kiszlinger H., Farkas J., Kover G., Nagy I. (2013). Selection for reproduction traits in Hungarian pig breeding in a two-way cross. Anim. Sci. Pap. Rep..

[51] Bennett G.L., Leymaster K.A. (1989). Integration of ovulation rate, potential embryonic viability and uterine capacity into a model of litter size in swine. J. Anim. Sci..

[52] Rosendo A., Iannuccelli N., Gilbert H., Riquet J., Billon Y., Amigues Y., Milan D., Bidanel J.P. (2012). Microsatellite mapping of quantitative trait loci affecting female reproductive tract characteristics in Meishan x Large White F2 pigs. J. Anim. Sci..

[53] Gaustad-Aas A.H., Hofmo P.O., Karlberg K. (2004). The importance of farrowing to service interval in sows served during lactation or after shorter lactation than 28 days. Anim. Reprod. Sci..

[54] Ziedina I., Jonkus D., Paura L. (2011). Genetic and phenotypic parameters for reproduction traits of Landrace sows in Latvia. Agric. Conspec. Sci..

[55] Koketsu Y., Dial G.D. (1998). Interactions between the associations of parity, lactation length, and weaning-to-conception interval with subsequent litter size in swine herds using early weaning. Prev. Vet. Med..

[56] Koketsu Y., Tani S., Iida R. (2017). Factors for improving reproductive performance of sows and herd productivity in commercial breeding herds. Porc. Health Manag..

[57] Knauer M.T., Hostetler C.E. (2013). U.S. swine industry productivity analysis, 2005 to 2010. J. Swine Health Prod..

[58] Belstra B.A., Diekman M.A., Richert B.T., Singleton W.L. (2005). Effects of lactation length and an exogenous progesterone and estradiol-17β regimen during embryo attachment on endogenous steroid concentration and embryo survival in sows. Theriogenology.

[59] Soede N.M., Langendijk P., Kemp B. (2011). Reproductive cycles in pigs. Anim. Reprod. Sci..

[60] Rhodes P.A., Liptrap R.M., Geissinger H.D. (1983). A correlative study of porcine endometrium and hormone levels during early lactation and the late luteal phase. Scan. Electron. Microsc..

[61] Bertoldo M.J., Holyoke P.K., Evans G., Grupen C.G. (2012). Seasonal variation in the ovarian function of sows. Reprod. Fertile. Dev..

[62] Tummaruk P. (2012). Effects of season, outdoor climate and photo period on age at first observed estrus in Landrace x Yorkshire crossbred gilts in Thailand. Livest. Sci..

